# Zoonotic transmission of *Chlamydia felis* from domestic cats; A case series of chronic follicular conjunctivitis in humans

**DOI:** 10.1016/j.nmni.2024.101412

**Published:** 2024-04-14

**Authors:** Laura Hughes, Stijn Visser, Edou Heddema, Nicole de Smet, Tim Linssen, Robert Jan Wijdh, Robert Huis in ’t Veld

**Affiliations:** a University Medical Centre Groningen, Department of Medical Microbiology, Groningen, the Netherlands; b University Medical Centre Groningen, Department of Ophthalmology, Groningen, the Netherlands; c Zuyderland Medical Centre, Department of Medical Microbiology, Sittard-Geleen, Heerlen, the Netherlands; d Zuyderland-Eyescan BV, Department of Ophthalmology, Zuyderland Medical Center, Sittard-Geleen, the Netherlands; e Huisartsenpraktijk Urmond, Urmond, the Netherlands

**Keywords:** *Chlamydia felis,* zoonosis, Follicular conjunctivitis, Sequence analysis

## Abstract

We present three cases of chronic follicular conjunctivitis resulting from the zoonotic transmission of *Chlamydia felis* from domestic cats. Our objective is to raise awareness regarding the potential zoonotic transmission of *Chlamydia felis* from domestic cats and describe the methodology for definitive pathogen identification using Polymerase Chain Reaction (PCR) and subsequent sequence analysis, a useful tool in the identification of these rare pathogens. We discuss the factors that could be contributing to the potential under-diagnosis of zoonotic *C. felis* infections and propose a treatment regime for cases of *C. felis*-related conjunctivitis.

## 1 Introduction

*Chlamydia felis* is an important cause of conjunctivitis, and sometimes respiratory tract infections, in domestic cats (*Felis catus*) [1,2]. It is a Gram-negative obligate intracellular bacterium and therefore cannot be cultured using standard media. As such, diagnosis of Chlamydiae relies on the use of molecular diagnostic tools including Polymerase Chain Reaction (PCR) [3].

In cats, *C. felis* infections are most common in young animals (less than one year old). Infected cats can shed *C. felis* in ocular and respiratory tract secretions and rectally for up to two months, during which time most domestic cats will show signs of conjunctivitis and/or rhinitis [4]. However, it is known that some cats remain persistently asymptomatically infected and shed *C. felis* for longer periods of time [5], posing a potential zoonotic risk. Transmission between cats occurs through direct, close contact because *C. felis* does not survive well in the environment. As such, cats kept in groups (e.g., shelters) are more susceptible to infection [4].

Most cases of chlamydial conjunctivitis in humans are caused by *Chlamydia trachomatis*. Less frequently, other Chlamydial species have been found to be the cause of conjunctivitis including *Chlamydia psittaci*, *Chlamydia pneumoniae* and *C. felis* [6]. To date, there are 6 cases of *C. felis* associated follicular conjunctivitis in humans documented in the literature, the first case being documented by Ostler et al. in 1969 [7]. Three of the case reports were in immunocompetent patients, one case was in a patient with Human Immunodeficiency Virus (HIV) and the immune status of the two other patients was not reported [3,[7], [8], [9], [10]].

Here we report three cases of follicular conjunctivitis in patients infected via zoonotic transmission from domestic cats and describe how the definitive identification of the pathogen was made by Polymerase Chain Reaction (PCR) and subsequent sequence analysis, a useful tool in the identification of these rare pathogens. We propose a treatment regime for cases of *C. felis*-related conjunctivitis.

## 2 Case report one

A 38-year-old female was referred to the ophthalmology out-patient clinic of a tertiary referral center in the Netherlands with a 3-month history of red, painful eyes. The patient reported severe irritation of both eyes, for which she had already received a single 1 goral dose of azithromycin, local tobramycin, dexamethasone and fluorometholone eye drops (corticosteroid) without success. During the 3-month period, the patient had been tested for Chlamydial infection by the referring ophthalmologist, each time with a negative result. The patient had no significant past medical history and reported no previous eye infections. The patient had intraocular lenses (Artiflex) and therefore did not wear contact lenses. None of the patient's family members, with whom she shared a household, reported any eye symptoms.

The patient reported that her cat had sneezed into her face prior to the onset of her symptoms.

Ophthalmological examination of the patient revealed a reduced visual acuity of 20/50 (0,4) in the left eye and a normal visual acuity of 20/16 (1,2) in the right eye. In the right eye a mild papillary conjunctivitis of the nasal inferior tarsus and a marked papillary conjunctivitis of the superior tarsus was observed. Substantial papillary and follicular conjunctivitis of the inferior tarsus and a substantial giant papillary conjunctivitis of the superior tarsus of the left eye was present. The cornea of the left eye was unaffected however the cornea of the right eye showed a slightly irregular epithelium.

Conjunctival swabs for bacterial culture grew commensal skin flora. A Gram stain revealed no leucocytes, bacteria, fungi, or yeasts. The patient's serum was tested for antibodies against *Chlamydia* species (*Chlamydia* serology is not specific and shows cross-reactivity with several species) using an enzyme-linked immunosorbent assay (Serion ELISA classic, Virion\Serion, Würzburg, Germany). The patient tested positive for *Chlamydia* spp. IgA and IgG, which was suggestive of a recent infection.

PCRs for bacterial and viral pathogens were performed on conjunctival swabs from the patient including a PCR for *Chlamydia psittaci* (cross-reactive with *C. felis*) due to the patient's history of a cat sneezing in her face.

A conjunctival swab from the patient was placed in Universal Transport Medium (UTM) (Copan Diagnostics Inc., California, United States of America). DNA isolation and real-time PCR for *Chlamydia pneumonia* and *Chlamydia psittaci* was performed on a 250 μL aliquot using the b-CAP real-time PCR assay (gene target: outer membrane protein A gene, *ompA*) according to the protocol described by the manufacturer (Biolegio, Veldhoven, The Netherlands) on a BD-MAX system (BD Molecular Diagnostics, New Jersey, United States of America).

The conjunctival swab from the patient tested positive for *C. psittaci* with a CT-value of 39.

The sample was then sent to the Department of Medical Microbiology of the Zuyderland Medical Center (ZMC) –the Dutch National Reference Center for zoonotic *Chlamydia* species infections, for subsequent confirmation and species determination of the Chlamydial strain.

A diagnostic real-time PCR directed at the conserved domain of the Major Outer membrane protein gene was performed. This PCR can also detect *C. abortus, C. felis* and *C. caviae*, although there are mismatches in the primers for *C. felis* [11].

The *Chlamydia* species real-time PCR amplifies a 98 base pair (bp) segment of the 23s rRNA gene using primer Chlamydia 23s forward (5′- GAA AAG AAC CCT TGT TAA GGG AG -3′) and primer Chlamydia 23s reverse (5′- ACC TCG CCG TTT ARC TTA ACT CC -3′) as previously described [12,13].

Sanger Sequence analysis was performed by big-dye terminator technology (Baseclear, Leiden).

**Fig. 1:**
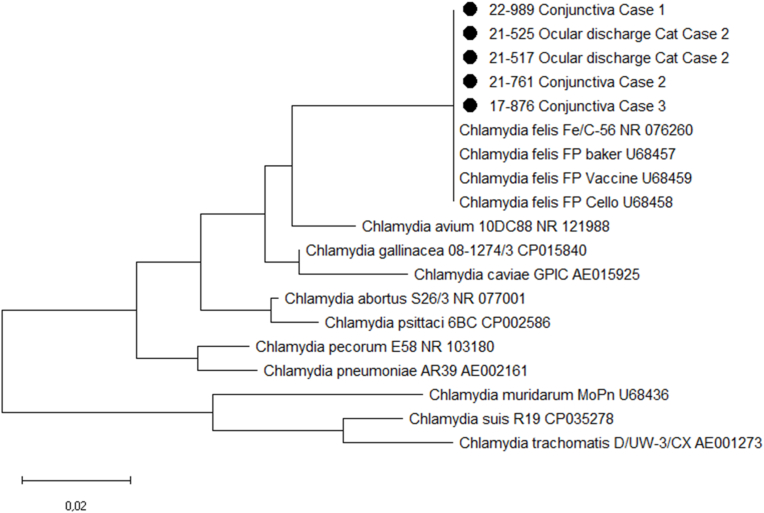
Phylogenetic tree of partial 23s rRNA gene sequences from *Chlamydia felis* isolates described in this case series compared with reference sequences derived from Genbank determined using P-distance. Nucleotide sequences were aligned (98 bp) and analyzed using MegaXv11 software. ● Samples related to this case series.

Sequence analysis of the PCR product identified the isolate as *C. felis*. A phylogenetic tree of the partial 23s rRNA gene sequences from the *Chlamydia felis* isolates described in this case series compared with reference sequences derived from Genbank was determined using P-distance (Fig. 1). Nucleotide sequences were aligned (98 bp) and analyzed using MegaXv11 software.

The patient was treated with doxycycline 100 mg twice a day for 28 days. After 28 days, the patient was reassessed. The patient's visual acuity had returned to the normal baseline of the patient and there was no evidence of follicular conjunctivitis. The patient's cat also tested positive for *C. felis* using the *Chlamydia* spp. PCR followed by sequence analysis.

## 3 Case report 2

A 34-year-old female consulted her general practitioner with a 10 day history of conjunctivitis of the left eye, with eyelid swelling and purulent ocular discharge. The patient reported no pain and no change to her visual acuity. She was empirically treated with chloramphenicol eye drops 4 mg/mL 3–4 drops per day for two weeks to no good effect. During the follow up visit, the patient complained of pain in her left eye. Ophthalmological examination revealed mild erythema of the sclera of the left eye, swelling of the superior tarsus and marked epiphora. Fluorescein staining of the eye showed no abnormalities of the cornea. Because of the lack of treatment response, infection with *Chlamydia trachomatis* was considered as a differential diagnosis. Conjunctival swabs were taken for PCR and the patient was prescribed a single oral dose of 1 g azithromycin and topical tetracycline eye ointment (10 mg/g) for two weeks. This treatment led to a marked improvement and full recovery within two weeks.

The conjunctival swabs were submitted to the *Zuyderland* Medical Center for a *Chlamydia trachomatis* PCR, but by mistake a *Chlamydia* spp. real-time PCR was requested instead of a *Chlamydia trachomatis* PCR (both tests are available at the ZMC). The *Chlamydia* spp. PCR, which can detect all currently known species, was positive and consecutive partial sequence analysis of the 23s gene (as described in case report 1) revealed the species to be *Chlamydia felis* (Fig. 1). On questioning it appeared that the patient owned 4 cats that had recently been treated for conjunctivitis by a veterinarian. Attempts to test her cats for *C. felis* were unsuccessful.

## 4 Case report 3

A 31-year-old female was referred to an ophthalmology out-patient clinic by her general practitioner with a 2-week history of a red left eye with a feeling of a foreign particle under the eyelid. Her past medical history was unremarkable. Under the assumption of an episcleritis, her general practitioner had treated her with prednisolone and chloramphenicol eyedrops. Ophthalmological examination of the patient revealed a slightly reduced visual acuity of 0.9 in the left eye and a normal visual acuity in the right eye. Hyperaemia and papillary and follicular conjunctivitis of the left eye were observed. The patient was prescribed Trafloxal eyedrops (ofloxacin 3 mg/mL) four times a day and ointment at night. Conjunctival swabs were taken for *Chlamydia trachomatis* PCR. Bacterial culture was not requested. By mistake, a *Chlamydia* spp. and *C. psittaci* multiplex real-time PCR was requested and gave a positive result (*C. psittaci*: Ct-value 36.5; *Chlamydia* spp.: Ct-value 32.5). Additional sequence analysis of the PCR product (as described in case report 1) identified the isolate as *C. felis* (Fig. 1). Based on this result antibiotic treatment was switched to doxycycline 100 mg once daily for 14 days with a loading dose of 200 mg on the first day of treatment. In addition, azithromycin eyedrops twice a day for three days were prescribed. Before symptom onset, the patient had cared for parrots owned by her parents for 2 weeks. As species determination was still ongoing, public health officials tested the birds for *C. psittaci* by PCR but they tested negative.

After 28 days the patient was reassessed. The patient had no further symptoms and her follicular conjunctivitis had resolved. *Chlamydia* spp. PCR's on conjunctival swabs from both eyes were negative at this time.

The patient reported that she had a cat, which had been sick with episodes of vomiting 6 weeks prior to the patient developing follicular conjunctivitis. Meanwhile she had managed to take a swab sample from early morning eye discharge from the cat. This was tested and found to be positive in the *Chlamydia* spp. PCR. Sequence analysis was performed as described in case report 1 and revealed the species to be *C. felis* (Fig. 1). At the time of testing the cat had no symptoms. The cat was seen by a veterinarian for the *C. felis* infection but a decision was made not to treat due to the cat being an asymptomatic carrier.

## 5 Discussion

Here we describe three cases of *C. felis*-associated papillary conjunctivitis over a period of 6 years from 2017 until 2022. *C. felis* is considered a rare cause of papillary conjunctivitis in humans, with only 6 cases reported in the literature over a period of 48 years prior to the three cases that we report here [3,[7], [8], [9], [10]]. Two from the three cases that we report here were detected by “mistake” when samples were screened for *Chlamydia* spp. with a *Chlamydia* spp. real-time PCR or tested for *C. pneumonia* and *C. psittaci* using the b-CAP RT-PCR assay, which gave a positive signal for *C. psittaci*. *C. felis* is known to be closely related to *C. psittaci* [14]. The reverse primer for *C. psittaci* in the b-CAP RT-PCR assay has only two mismatches compared with *C. felis* explaining why the *C. felis* isolates from two cases gave a positive result for *C. psittaci*, albeit at higher Ct-values.

There are no diagnostic microbiology laboratories in the Netherlands that have a specific diagnostic test for *C. felis*. In the three cases that we report, *C. felis* was detected due to a positive result for *C. psittaci* or *Chlamydia* spp. in a multiplex real-time PCR. Sequence analysis was then performed on the PCR product from case one because the clinical microbiologist suspected *C. felis* infection after ascertaining that the patient had a history of exposure to cats. In cases 2 and 3, only the final identification of *C. felis* pointed towards patient's cats as the source of infection. It is therefore important to raise awareness among ophthalmologists, general practitioners and clinical microbiologists that *C. felis* should be considered in the differential diagnosis in cases of follicular conjunctivitis with a history of exposure to cats. This should be confirmed with a positive PCR for *C. psittaci* (cross-reacting with *C. felis*) or *Chlamydia* spp., followed by subsequent targeted sequencing, or a specific *C. felis* PCR as described by Wons et al. [3].

The prevalence of *C. felis* in domestic cats in the Netherlands is unknown. A recent survey in Switzerland of 309 stray cats and 86 pet cats revealed a *C. felis* prevalence of 18.1 % and 11.6 % in stray and pet cats respectively [15]. In 2019 a similar cross-sectional survey of 57 stray/shelter cats and 83 pet cats in Slovakia revealed a *C. felis* prevalence of 33.3 % in stray/shelter cats and 10 % in pet cats that were allowed to go outside regularly [16]. In both surveys, significantly higher rates of *C. felis* infection were found in cats displaying signs of conjunctivitis but there were also asymptomatic carriers identified [15,16] and a study of *C. felis* in cats in Italy also revealed a prevalence of 3 % in asymptomatic healthy cats [17].

Vaccines against *C. felis* for use in domestic cats are available but *C. felis* is not included in the “standard” cat vaccination scheme in the Netherlands, which includes vaccines against feline panleukopenia virus, feline calcivirus and feline herpes virus. Furthermore, feline Chlamydial vaccines do not provide complete protection [4]. Therefore the number of cats vaccinated against *C. felis* in the Netherlands is likely to be low.

There are no treatment guidelines for *C. felis-*associated follicular conjunctivitis. The current first choice antibiotic treatment for *C. trachomatis*-associated conjunctivitis in the Netherlands is a single 1 g oral dose of azithromycin [18]. Of the three patients described here, two patients were treated with this antibiotic regimen (case 1 and case 2). In case 1, the treatment was given initially by the referring ophthalmologist however it was clinically unsuccessful. In case 2, treatment with azithromycin appeared to be clinically successful however, azithromycin was given in combination with tetracycline eye ointment after the patient had been treated with chloramphenicol eye drops for two weeks. Treatment failure up to 17 % with this antibiotic regimen has been reported for several infections caused by *C. trachomatis*, especially in rectal infections [[19], [20], [21], [22], [23]] and the treatment of trachoma in young children. Azithromycin is no longer the first-choice antibiotic for these infections, instead 100 mg doxycycline orally twice a day for between 7 and 21 days is recommended and has been shown to be more effective [24]. Therefore, a patient presenting with chronic conjunctivitis who has recently been treated with 1 g azithromycin should be evaluated carefully before ruling out chlamydial infection. In two of the three cases described (1 and 3), oral doxycycline was part of the treatment regime that resulted in the successful treatment of *C. felis*-associated follicular conjunctivitis. The dose of doxycycline used in case 1 was 100 mg twice a day for 28 days and a shorter course was used in case 3 of 100 mg once a day for 14 days with a loading dose of 100 mg twice a day on the first day. A prolonged course of doxycycline was also required to eradicate infection in two cases of *C. felis* associated follicular conjunctivitis described by Hartley et al. [10] and Wons et al. [3] This would suggest that a longer course of doxycycline (a loading dose followed by 14–28 days) should be considered as a treatment regime in cases of *C. felis* associated conjunctivitis.

In conclusion, in cases of chronic follicular conjunctivitis, patients should be questioned about exposure to cats and *C. felis* should be considered in the differential diagnosis and confirmed with a positive PCR for *C. psittaci* or *Chlamydia* spp., and subsequent targeted sequencing or, if available, specific *C. felis* PCR.

## Ethics statement

Informed consent was obtained from all patients.

## Funding

Species determination by sequence analysis was financially supported by a grant from the 10.13039/501100007192RIVM, the Dutch National Institute for Public Health and the Environment.

## CRediT authorship contribution statement

**Laura Hughes:** Writing – original draft, Visualization, Investigation, Formal analysis, Data curation. **Stijn Visser:** Writing – original draft, Visualization, Investigation, Formal analysis. **Edou Heddema:** Writing – review & editing, Methodology, Formal analysis, Data curation. **Nicole de Smet:** Writing – review & editing, Formal analysis, Data curation. **Tim Linssen:** Writing – review & editing, Formal analysis, Data curation. **Robert Jan Wijdh:** Writing – review & editing, Supervision, Investigation, Conceptualization. **Robert Huis in ’t Veld:** Writing – review & editing, Supervision, Methodology, Investigation, Conceptualization.

## Declaration of competing interest

All authors declare that they have no conflicts of interest.
